# Herpes simplex virus blocks host transcription termination via the bimodal activities of ICP27

**DOI:** 10.1038/s41467-019-14109-x

**Published:** 2020-01-15

**Authors:** Xiuye Wang, Thomas Hennig, Adam W. Whisnant, Florian Erhard, Bhupesh K. Prusty, Caroline C. Friedel, Elmira Forouzmand, William Hu, Luke Erber, Yue Chen, Rozanne M. Sandri-Goldin, Lars Dölken, Yongsheng Shi

**Affiliations:** 1 Department of Microbiology and Molecular Genetics, School of Medicine, University of California, Irvine, Irvine, CA 92697 USA; 2 Institute for Virology and Immunobiology, Julius-Maximilians-University Würzburg, Würzburg, Germany; 3 Institute of Informatics, Ludwig-Maximilians-Universität München, München, Germany; 4 Institute for Genomics and Bioinformatics, University of California, Irvine, Irvine, CA 92697 USA; 5 Department of Computer Science, University of California, Irvine, Irvine, CA 92697 USA; 6 Department of Biochemistry, Molecular Biology, and Biophysics, College of Biological Sciences, University of Minnesota, Saint Paul, MN 55018 USA; 7 Helmholtz Institute for RNA-based Infection Research, Würzburg, Germany

**Keywords:** Herpes virus, Virus-host interactions, RNA metabolism, Transcription

## Abstract

Infection by viruses, including herpes simplex virus-1 (HSV-1), and cellular stresses cause widespread disruption of transcription termination (DoTT) of RNA polymerase II (RNAPII) in host genes. However, the underlying mechanisms remain unclear. Here, we demonstrate that the HSV-1 immediate early protein ICP27 induces DoTT by directly binding to the essential mRNA 3’ processing factor CPSF. It thereby induces the assembly of a dead-end 3’ processing complex, blocking mRNA 3’ cleavage. Remarkably, ICP27 also acts as a sequence-dependent activator of mRNA 3’ processing for viral and a subset of host transcripts. Our results unravel a bimodal activity of ICP27 that plays a key role in HSV-1-induced host shutoff and identify CPSF as an important factor that mediates regulation of transcription termination. These findings have broad implications for understanding the regulation of transcription termination by other viruses, cellular stress and cancer.

## Abstract (web summary)

Herpes simplex virus-1 (HSV-1) infection disrupts transcription termination (DoTT) of host genes, but underlying mechanisms are unclear. Here, Wang et al. show that the HSV-1 immediate early protein ICP27 induces DoTT through interaction with the mRNA 3’ processing factor CPSF and disruption of the processing complex.

## Introduction

RNA polymerase II (RNAPII) transcription termination is an essential step in eukaryotic gene expression, but its mechanism remains elusive^1–4^. Two models have been proposed, the allosteric model and the torpedo model. In both models, poly(A) site (PAS) and the mRNA 3′ processing machinery are essential^5–10^. Most mammalian PAS consists of an A(A/U)UAAA hexamer, a U/GU-rich downstream element, and various auxiliary sequences^11^. These RNA sequences recruit the mRNA 3′ processing factors CPSF, CstF, and CFIm, CFIIm and the poly(A) polymerase to assemble the mRNA 3′ processing complex. Within this complex, mRNA 3′ processing takes place in two steps, an endonucleolytic cleavage and the subsequent addition of a poly(A) tail. Many but not all mRNA 3′ processing factors are required for transcription termination^12^.

Recent studies have revealed that RNAPII transcription termination is regulated. Through nascent RNA sequencing, we first demonstrated that lytic herpes simplex virus-1 (HSV-1) infection induces widespread disruption of transcription termination (DoTT) in host genes^13^. The Steitz group subsequently reported that osmotic stress induces transcription downstream of human genes (DoGs)^14^. Further studies demonstrated that influenza virus infection and other cellular stresses, such as heat shock, also cause widespread DoTT and that the genes affected by virus- and stress-induced DoTT are highly correlated^15^. Importantly, pervasive DoTT was observed in renal cell carcinoma due to the loss of the histone methyltransferase SETD2 (ref. ^16^). The mechanisms of virus- or stress-induced DoTT/DoGs, however, remain poorly defined.

HSV-1 is a DNA virus that causes a number of diseases ranging from painful skin lesions to encephalitis^17^. Like other viruses, HSV-1 hijacks host factors to facilitate productive infection while efficiently shutting down host gene expression. Its tegument protein VP16 recruits the host transcription machinery to express immediate early genes, including ICP0, ICP4, ICP27, and ICP47. ICP4, in turn, activates the transcription of early and late genes. ICP27 regulates viral gene expression through several mechanisms, including splicing^18^, 3′ processing^19,20^, and mRNA export^21,22^. Earlier studies provided evidence that ICP27 inhibits splicing^18^. As most viral genes are intronless, ICP27-mediated splicing inhibition was proposed to specifically block host gene expression. Recent transcriptomic analyses, however, revealed that neither HSV-1 infection nor ICP27 overexpression globally inhibits splicing, but rather modulates alternative splicing of a subset of cellular genes^13,19^, suggesting that splicing regulation is not a major mechanism for HSV-1-induced host shutoff.

Here we dissect the molecular mechanism of HSV-1-induced DoTT. Our data show that ICP27 blocks transcription termination of host genes by inhibiting mRNA 3′ processing. Meanwhile ICP27 can act as a sequence-dependent activator of mRNA 3′ processing for viral transcripts. These results reveal a critical role for ICP27 in mediating host shut-off during HSV-1 infection.

## Results

### The role of immediate early proteins in HSV-1-mediated DoTT

**Fig. 1:**
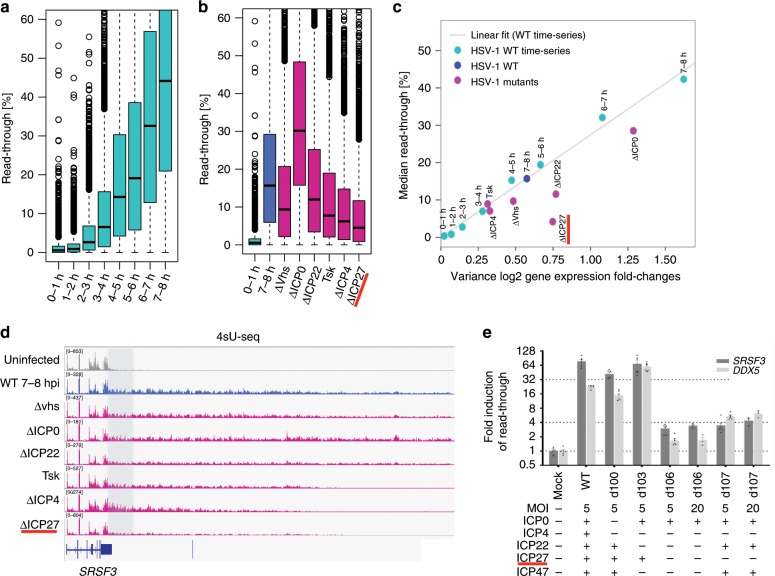
ICP27 is a major contributor for HSV-1-induced disruption of transcription termination. **a** Boxplots showing the distribution of read-through at different time points post-infection during HSV-1 wild-type infection (time-course). **b** Boxplots showing the distribution of read-through in cells infected with wild-type and various mutant viruses at 7–8 h (WT, Δvhs, ΔICP4, ΔICP27) and 11–12 h (ΔICP22, ΔICP0) post-infection. Please note that read-through at 7–8 h post-infection shown here is from a repeat experiment performed together with the mutant viruses. Read-through at 0–1 h post-infection from the WT time-course is also shown. **c** Median read-through values for each condition and time-point are plotted against the variance in log2 gene expression (=gene FPKM) fold-changes. The gray curve indicates the result of linear fit on all HSV-1 infection time-points from the time-course experiment. **d** Mapped 4sU-seq reads for *SRSF3* in cells infected with wild-type or various mutant HSV-1 strains. The region where transcription termination occurs in mock-infected cells is shaded. **e** Primary human fibroblasts infected were infected for 8 h with mutant viruses lacking various immediate early genes. The genes that are still expressed by the individual mutants as well as the employed multiplicity of infection are indicated in the table below the graph. Read-through transcription was quantified by qRT-PCR data and plotted as mean ± s.e.m. (*n* = 5).

Our previous transcriptome analysis of HSV-1-infected cells detected widespread DoTT in host genes^13^. To characterize the timing of DoTT, we monitored transcriptional activities at different time points post-infection by 4-thiouridine (4sU) pulse-labeling and high-throughput sequencing (4sU-seq)^13^. Significant DoTT was observed as early as 2 h post-infection (h.p.i.) and steadily increased thereafter (Fig. 1a). To identify the responsible viral gene(s), we infected primary human fibroblast cells with mutant viruses lacking key viral regulators (immediate early genes and vhs) and quantified DoTT by 4sU-seq. While knockout mutants of ICP0 and ICP22 as well as the key viral host shut-off protein vhs still induced DoTT, this was substantially reduced upon infection with viruses deficient for ICP4 (ΔICP4 or TsK, a temperature-sensitive mutant of ICP4) or ICP27 (ΔICP27) (Fig. 1b). Among these viral genes, deletion of ICP27 resulted in the greatest decrease in DoTT (Fig. 1b). Previously, we observed a strong linear correlation between DoTT and the variance in cellular mRNA expression changes during infection^15^. This correlation was essentially maintained for the mutant viruses including Δvhs, ΔICP22, ΔICP0 as well as ΔICP4 and TsK (Fig. 1c). DoTT induced by infection with the latter two mutant viruses matched DoTT observed 3–4 h.p.i. with wild-type (WT) HSV-1. In contrast, DoTT for ΔICP27 was substantially reduced compared to the observed alterations in host gene expression infected with the WT HSV-1 (Fig. 1c), suggesting that ICP27 plays a major role in HSV-1-induced DoTT. Two specific examples of DoTT in cells infected with the various mutant viruses are shown in Fig. 1d (*SRSF3*) and Supplementary Fig. 1 (*DDX5*). In contrast to uninfected cells, transcription continued over 30 kilobases (kb) downstream of the PAS in both genes. While DoTT was observed for all mutant viruses including ΔICP4, TsK and ΔICP27, read-through transcription protruded substantially less in ΔICP27 infection. These data suggest that multiple factors contribute to HSV-1-induced DoTT and that ICP27 plays a major role in this process.

In absence of ICP4, viral gene expression is restricted to the four remaining immediate early genes (ICP0, ICP22, ICP27, and ICP47). To directly assess the role of ICP27 in DoTT, we employed a range of deletion mutants that lack ICP4 in combination with various other immediate early genes^23,24^. We quantified DoTT for two marker genes (*SRSF3* and *DDX5*) by qRT-PCR upon infection of primary human fibroblasts with the respective mutant viruses (Fig. 1e). While deletion of ICP4 only resulted in a modest (~2-fold) reduction in read-through, knockout of ICP27 significantly reduced read-through transcription by > 20-fold. This was particularly prominent when comparing the two mutants d103 (expressing only ICP0 and ICP27) and d106 (expressing only ICP0), which only differ by the expression of ICP27. Importantly, DoTT could not be rescued by increasing the dose of infection of both d106 and d107 from a multiplicity of infection (MOI) of 5–20, thereby excluding dose effects on the observed differences. We conclude that ICP27 mediates HSV-1 induced DoTT.

### ICP27 is sufficient to induce DoTT

**Fig. 2:**
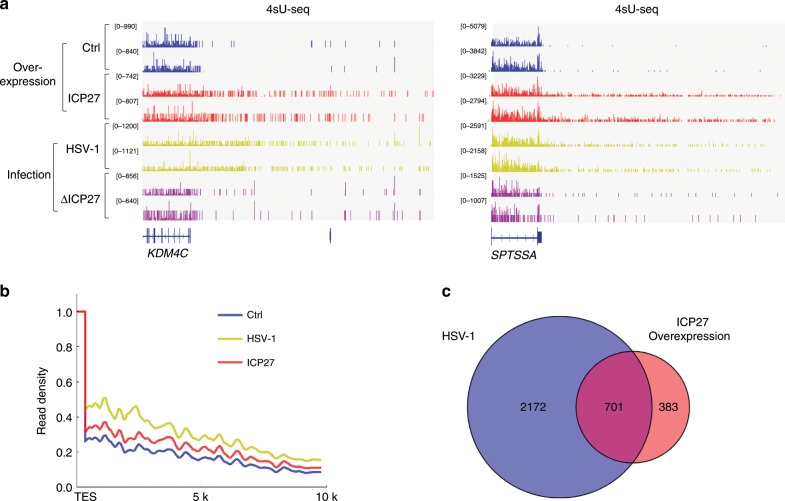
ICP27 is sufficient to inhibit RNAPII transcription termination. **a** 4sU-seq tracks of *KDM4C* and *SPTSSA* genes in cells transfected with vector or an ICP27-expressing plasmid. For comparison, 4sU-seq tracks for cells infected with WT or ΔICP27 HSV-1 were also included. Two replicates for each condition are shown. **b** Metagene analysis of 4sU-seq signals at the transcript end site (TES) in cells transfected with vector or an ICP27-expressing plasmid or infected with HSV-1. **c** Venn diagram showing the overlap of genes with significant termination defects in cells infected with HSV-1 or transfected with ICP27 overexpression.

We next asked whether ectopic expression of ICP27 alone was sufficient to induce DoTT. To this end, we expressed ICP27 in HeLa cells by transient transfection and performed 4sU-seq. Interestingly, we observed significant DoTT upon transfection of ICP27 but not control (empty vector) in many host genes, including *KDM4C* and *SPTSSA* (Fig. 2a), similar to HSV-1-induced DoTT. Genome-wide analysis confirmed extensive transcriptional activity downstream of the normal transcript end site (TES) in ICP27-expressing cells (Fig. 2b), albeit that the 4sU-seq signal density was significantly less compared to that in HSV-1 infected cells (Fig. 2b). In addition to the extent of DoTT, we also analyzed the pattern of genes that displayed DoTT induced by HSV-1 or transient transfection of ICP27. Of note, 65% (701 genes) of genes with significant DoTT (5-fold change in 4sU-seq signal downstream/upstream of PAS) in ICP27-expressing cells also displayed similar defect in HSV-1-infected cells (Fig. 2c). Together, these results strongly suggest that ICP27 by itself is sufficient for inhibiting RNAPII transcription termination and is a major contributor of HSV-1-induced DoTT.

### ICP27 interacts specifically with CPSF

**Fig. 3:**
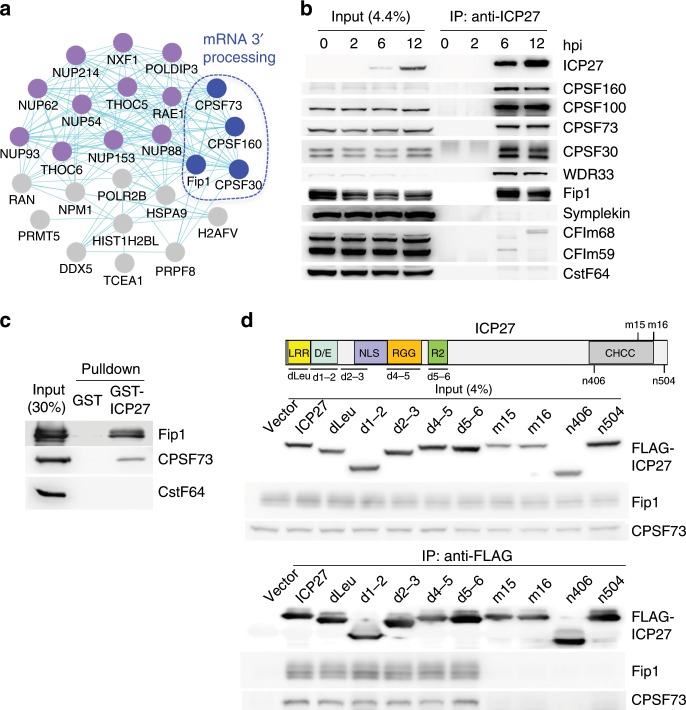
ICP27 directly interacts with CPSF and the mRNA 3′ processing machinery. **a** A network plot of the top ICP27-associated host factors. The purple dots are factors with known functions in mRNA export and the blue dots are mRNA 3′ processing factors. The plot was based on STRING. **b** Cells infected with WT HSV-1 (KOS) were harvested at different time points post-infection and subjected to immunoprecipitation with an anti-ICP27 antibody. Input and IP samples were analyzed by western blotting. **c** In vitro pulldown assays with GST-ICP27 and recombinant Fip1, CPSF73, and CstF complex. The input and pulldown samples were analyzed by western blotting. **d** Top panel: a diagram of known domains in ICP27 protein and the regions deleted in the mutant ICP27 proteins are marked below. Lower panel: WT or various mutant Flag-tagged ICP27 were expressed in cells via transient transfection and IP was performed with anti-Flag antibody. The input and IP samples were analyzed by western blotting. Source data are provided as a Source Data file.

To understand how ICP27 inhibits RNAPII transcription termination, we first identified the host factors that are associated with ICP27 during HSV-1 infection. We immunoprecipitated ICP27 from WT HSV-1-infected HeLa cells and identified the precipitated proteins by mass spectrometry analysis. Lysates from HeLa cells infected with an ICP27 null mutant (27LacZ) served as controls. All lysates were treated with RNase A/T1 prior to Immunoprecipitation (IP) to facilitate detection of protein–protein interactions. Proteins that were specifically identified in WT- but not in 27lacZ virus-infected cells were considered as ICP27-associated proteins (Supplementary Fig. 2a and Supplementary Data 1). Among the co-precipitated proteins were PABP1 and 11 cellular proteins with known functions in mRNA export (purple dots in Fig. 3a), consist with the known function of ICP27 in mRNA export^21^. Interestingly, we also identified four subunits of the CPSF complex (CPSF73, Fip1, CPSF160, and CPSF30) as ICP27-associated factors (blue dots, Fig. 3a). As mentioned earlier, mRNA 3′ processing is required for RNAPII transcription termination^1–4^. Knockdown of CPSF subunits, such as CPSF73, has been shown to induce DoTT^25,26^. Thus, we hypothesized that ICP27 inhibits transcription termination via its interaction with CPSF.

To test this hypothesis, we characterized the interactions between ICP27 and CPSF. We first validated the interactome mapping results by immunoprecipitating ICP27 following HSV-1 infection and analyzing the precipitated proteins by western blotting. Consistent with our mass spectrometry results, CPSF subunits including CPSF160, CPSF100, CPSF73, CPSF30, Wdr33, and Fip1, all co-precipitated with ICP27 (Fig. 3b). By contrast, other mRNA 3′ processing factors, including CstF and CFIm subunits, were not detected at significant levels (Fig. 3b), suggesting that ICP27 specifically interacts with the CPSF complex. Symplekin is a core component of the mRNA 3′ processing machinery and it associates with both CPSF and CstF complex^27,28^. Intriguingly, little to no symplekin was detected in ICP27 IP samples (Fig. 3b). We next determined if the interactions between ICP27 and CPSF required any other viral factors. To this end, we immunoprecipitated ICP27 upon transient transfection in 293T cells and performed mass spectrometry and western blotting analysis of the IP samples. Consistent with our IP analyses of HSV-1-infected cells, ectopically expressed ICP27 co-precipitated CPSF subunits, but not other mRNA 3′ processing factors (Supplementary Fig. 2b and Supplementary Data 1). Furthermore, symplekin again did not co-precipitate with ICP27 (Supplementary Fig. 2b), indicating that ICP27 and symplekin may bind to CPSF complex in a mutually exclusively manner. Together these results demonstrate that ICP27 specifically interacts with CPSF and that this interaction does not require other viral proteins.

We next determined which CPSF subunit(s) directly interact with ICP27. We purified recombinant GST-ICP27 and various mRNA 3′ processing factors and performed GST pulldown assays. Among the tested 3′ processing factors, GST-ICP27 pulled down Fip1 and CPSF73 of the CPSF complex, but not CstF (Fig. 3c), indicating that ICP27 directly interacts with multiple subunits of the CPSF complex. ICP27 contains a number of known functional domains, including the Leucine-rich region, the RGG box, and the C-terminal zinc finger domain (Fig. 3d, top panel)^29^. In order to map the domain(s) that mediate CPSF interaction, we expressed Flag-tagged WT or various mutant ICP27 by transient transfection, and performed IP with anti-Flag antibodies. The mutant ICP27 included deletion mutants in which specific domain/regions were deleted, such as dLeu, d1-2, d2-3, d4-5, and d5-6 (Fig. 3d). In addition, m15 is a substitution of PG to LE at amino acids 465, 466, and m16 is a C to L substitution at residue 488^30^. n406 has a stop codon in all three reading frames at amino acid 406 and n504 has a stop codon in all three reading frames at amino acid 504^31^. Interestingly, the CPSF subunits, Fip1 and CPSF73, were efficiently co-precipitated by ICP27 mutants lacking the N-terminal region, the NLS, and the RGG box. However, little or no Fip1 or CPSF73 were co-precipitated by ICP27 mutants in which the C-terminal regions were deleted or mutated (m15, m16, n406, and n504, Fig. 3d). We conclude that the C-terminal domain of ICP27 is necessary for mediating interactions with CPSF.

### ICP27 represses mRNA 3′ end processing

**Fig. 4:**
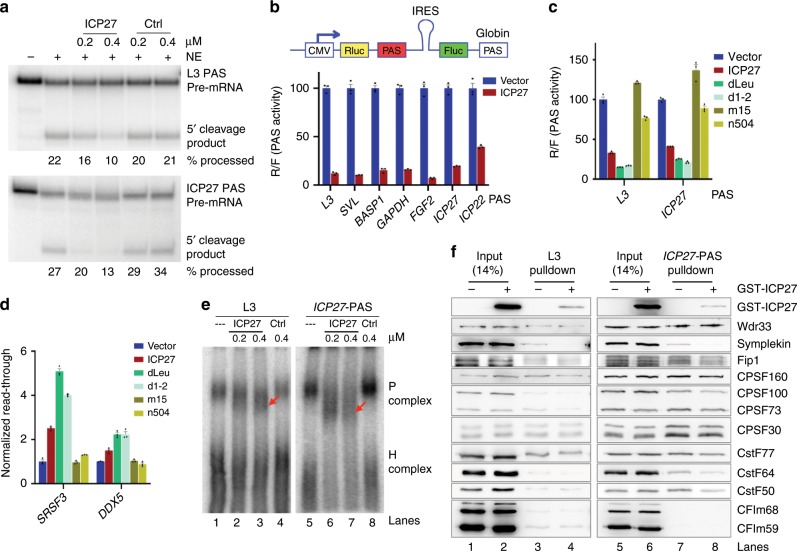
ICP27 inhibits mRNA 3′ processing. **a** In vitro cleavage assay with HeLa nuclear extract (NE) using L3 (top panel) or *ICP27* (lower panel) PAS pre-mRNA substrate. 5′ cleavage product is marked. Different amounts of recombinant MBP-ICP27 (labeled as ICP27) or MBP-MS2 (Ctrl) were added to NE. **b** Dual luciferase reporter assay. The top panel is a diagram for pPASPORT vector and the site where the PAS to be tested is inserted is marked as PAS (in a red box). Seven distinct PASs were cloned into pPASPORT, co-transfected with a vector or ICP27-expression plasmid. The Rluc/Fluc ratio, which measures cleavage/polyadenylation efficiency at the test PAS, are plotted as mean ± s.d. **c** pPASPORT assays with L3 and ICP27 PAS. Empty vector, ICP27 or its various mutants were co-expressed with the pPASPORT constructs and renilla/firefly luciferase ratio was plotted as mean ± s.d. **d** Empty vector, ICP27 or its various mutants were transiently expressed and transcription read-through at the *SRSF3* and *DDX5* genes were measured by qRT-PCR and plotted as mean ± s.d. **e** In vitro cleavage/polyadenylation reactions (similar to **a**) were resolved on a native gel and visualized by phosphorimaging. The mRNA 3′ processing complex (P complex) and the heterogeneous complex (H complex) are marked. **f** 3MS2-L3 or 3MS2-ICP27 PAS RNA substrates are incubated under cleavage/polyadenylation conditions and the assembled RNP complexes were pulled down by using amylose beads and analyzed by western blotting. Source data are provided as a Source Data file.

To test whether ICP27 affects mRNA 3′ processing, we performed in vitro 3′ processing assays. We incubated recombinant ICP27 with nuclear extract (NE) from HeLa cells and assessed mRNA 3′ cleavage of L3, a commonly used PAS RNA derived from adenovirus major late transcript. MBP-MS2, another RNA-binding protein, served as a control. Interestingly, ICP27 inhibited mRNA 3′ cleavage of L3 in a dose-dependent manner while MBP-MS2 had no effect (Fig. 4a). We also tested several other human and viral PASs using the same assay and found that ICP27 inhibited the cleavage of all tested PASs including the PAS of the ICP27 gene itself (Fig. 4a, lower panel, and Supplementary Fig. 3a). These data demonstrate that ICP27 inhibits mRNA 3′ processing in vitro.

To test the impact of ICP27 on mRNA 3′ processing in living cells, we took advantage of a dual luciferase reporter pPASPORT (Fig. 4b)^32^. In this reporter, *Renilla* (*Rluc*) and *Firefly* luciferase (*Fluc*) genes are expressed from a bicistronic mRNA in which translation of the downstream *Fluc* gene is driven by an internal ribosomal entry site (IRES). A candidate PAS to be tested is inserted between the two luciferase genes. If cleavage/polyadenylation occurs efficiently at the candidate PAS, only Rluc is expressed. In contrast, inefficient 3′ processing will lead to transcription read-through and termination at the strong downstream β-globin PAS, thereby resulting in the expression of both Rluc and Fluc. The Rluc/Fluc ratio thus provides a quantitative measurement of RNA cleavage/polyadenylation efficiency at the candidate PAS^32–34^.

Consistent with our in vitro data, expression of ICP27 significantly suppressed mRNA 3′ processing of all seven different PASs (three human, four viral) we tested (Fig. 4b). As ICP27 is known to regulate mRNA export and translation^22,35^, we wanted to determine if the effect of ICP27 observed in our reporter assay was due to its direct role in regulating mRNA 3′ processing. To this end, we isolated RNAs from the reporter assays and performed 3′ Rapid Amplification of cDNA Ends (RACE) (Supplementary Fig. 3b). In this assay, the two RNA isoforms expressed from the pPASPORT construct were amplified into two distinct PCR products in the same reaction. In the absence of ICP27, both RNA isoforms were detected (Supplementary Fig. 3b, lanes 1, 3, and 5). Co-expression of ICP27, however, led to a significant decrease in the shorter RNA isoform containing only Rluc (Supplementary Fig. 3b, lanes 2, 4, and 6), suggesting that ICP27 inhibited mRNA 3′ processing at the upstream PAS. These results demonstrate that the inhibitory effect of ICP27 observed in our reporter assay (Fig. 4b) was due to a direct role in regulating mRNA 3′ processing and not caused by other indirect effects. We conclude that ICP27 broadly inhibits mRNA 3′ processing. It is important to note that not only cellular but also the two HSV-1 viral PASs (of ICP27 and ICP22) that we tested were inhibited by ICP27, suggesting that the core PAS (from −100 nucleotides (nt) to +100 nt relative to the cleavage site) alone is not sufficient for ICP27 to distinguish between viral and host PASs.

We next mapped the domain/regions of ICP27 that are necessary for inhibiting mRNA 3′ processing. We over-expressed the WT or various mutant ICP27 and checked their effect on mRNA 3′ processing using the reporter assay. Our results demonstrate that mutant ICP27 with N-terminal deletions, such as dLeu and d1-2, inhibited mRNA 3′ processing to a similar degree as the WT ICP27 while the C-terminal mutants (m15 and n504) lost this ability (Fig. 4c). Additionally, we also compared the activities of the WT and mutant ICP27 in inhibiting host gene transcription termination by qRT-PCR. Consistent with the reporter assay results, the N-terminal deletion mutants of ICP27 strongly induced DoTT of host genes, similar to the WT ICP27. By contrast, the C-terminal mutants had no significant effect (Fig. 4d). Together these data suggest that the C-terminal domain of ICP27 is required for its interactions with CPSF and its inhibition of mRNA 3′ processing and transcription termination.

### ICP27 interferes with mRNA 3′ processing complex assembly

To assess which step of mRNA 3′ processing ICP27 interferes with, we first monitored the assembly of the mRNA 3′ processing complex, the earliest step of mRNA 3′ processing^11^. L3 or ICP27 PAS-containing RNAs were incubated with NE under cleavage/polyadenylation conditions and then resolved by native PAGE. In the absence of ICP27 (Fig. 4e, lanes 1 and 5), the mRNA 3′ processing complex (P complex) efficiently assembled on both RNA substrates. In the presence of ICP27, however, the P complex migrated faster on the gel and appeared more diffused (Fig. 4e, compare lanes 2–3 to lane 1 and compare lanes 6–7 to lane 5) although complex formation (overall signals) seemed unaffected. The control protein, MBP-MS2, had no effect. This suggests that ICP27 induces the assembly of an aberrant mRNA 3′ processing complex that may be compositionally and/or conformationally distinct from the normal complex.

To directly compare the composition of the mRNA 3′ processing complexes assembled in the presence or absence of ICP27, we purified these complexes using an RNA affinity approach^28,36^. Briefly, PAS-containing RNAs were fused to three tandem copies of the MS2 hairpin (the MS2 hairpins are ~60 nt upstream of the cleavage site). These RNA substrates were first incubated with MBP-MS2, which binds to the MS2 hairpins, and then with NE to allow for the assembly of mRNA 3′ processing complexes. These complexes were then pulled down by using amylose beads and analyzed by western blotting. By comparing the mRNA 3′ processing complexes assembled on L3 and ICP27 PAS with or without recombinant ICP27 protein (Fig. 4f), we confirmed that ICP27 associates with the mRNA 3′ processing complex (Fig. 4f, lanes 4 and 8). Furthermore, nearly all mRNA 3′ processing factors assembled at similar levels in the mRNA 3′ processing complex in the presence or absence of ICP27 (Fig. 4f, compare lanes 3 and 4 or 7 and 8). However, symplekin and CstF64 seemed to display a modest decrease (Fig. 4f, compare lanes 3 and 4 or 7 and 8). Together with our earlier observation that the ICP27-associated CPSF complex lacks symplekin (Fig. 3b), these results indicate that ICP27 interaction with CPSF prevents its stable association with symplekin and CstF. Furthermore, when we resolved the affinity purified P complexes assembled in the absence or presence of ICP27 by glycerol gradient sedimentation, we observed that the P complexes assembled in the presence of ICP27 failed to reach higher density fractions and spread out into more gradient fractions (Supplementary Fig. 4). This is consistent with our previous observation that the P complex assembled in the presence of ICP27 not only migrated faster but also appeared more diffuse on native gels (Fig. 4e). We conclude that ICP27 does not block the assembly of mRNA 3′ processing complex but rather interferes with mRNA 3′ processing by preventing the assembly of a functional mRNA 3′ processing complex.

### ICP27 activates viral mRNA 3′ processing

**Fig. 5:**
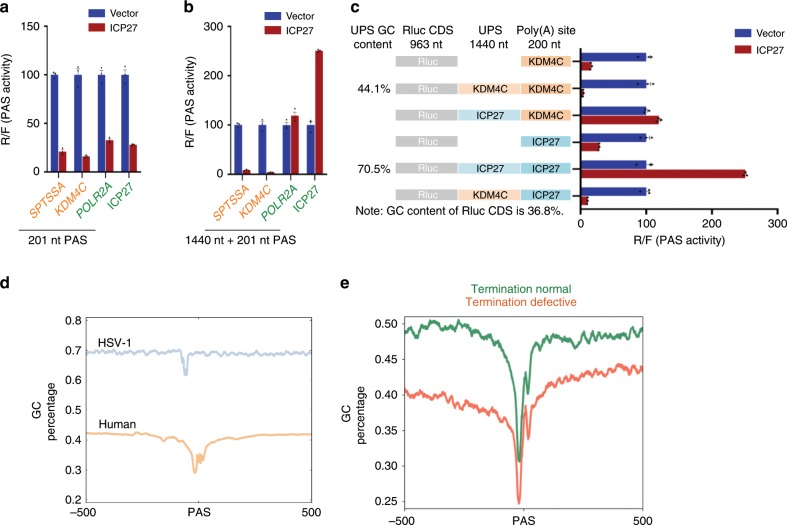
ICP27 is a sequence-dependent activator of mRNA 3′ processing. The core sequences (−100 nt to +100 nt (**a**)) or the extended sequences (core plus 1440 nt upstream sequences (**b**)) of four PASs, including *SPTSSA* and *KDM4C* (showed DoTT in HSV-1-infected cells) and *POLR2A* and *ICP27* (no DoTT), were cloned into pPASPORT. These reporters were co-transfected with vector or an ICP27-expressing plasmid and mRNA 3′ processing activities (Rluc/Fluc) were plotted as mean ± s.d. **c** The core and extended PAS of *KDM4*C and *ICP27* as well the chimeric PASs between the two PASs were co-transfected with vector or an ICP27-expressing plasmid and the PAS activities (Rluc/Fluc) were plotted as mean ± s.d. The GC contents of the upstream sequences of both PASs are marked on the left. **d** The GC content at HSV-1 and human PASs. **e** The GC contents of PASs of host genes with significant DoTT (red) and without DoTT (green). Reporter assays in **a**, **b**, and **c** were performed at the same time under the same conditions and were displayed in multiple panels for comparisons.

HSV-1 induces DoTT in thousands of host genes, while transcription termination for HSV-1 as well as some host genes remains mostly unaffected^13^. However, ICP27 inhibits mRNA 3′ processing of all PASs that we tested, including viral PASs (Fig. 4 and Supplementary Fig. 3). To understand the molecular basis for the specificity of HSV-1-induced DoTT, we selected four PAS for further studies. These included two cellular PASs displaying strong HSV-1 induced DoTT (*SPTSSA* and *KDM4C*, see Fig. 2a). In addition, we included a host PAS (*POLR2A*) and a viral PAS (ICP27), both of which showed normal transcription termination in HSV-1-infected cells (Supplementary Fig. 5). When we tested the core sequences of these PASs (from −100 nt to +100 nt relative to the cleavage site) in the pPASPORT assays, we again observed strong inhibition of mRNA 3′ processing by ICP27 (Fig. 5a). As the core PAS sequences were apparently not sufficient to explain the specificity of ICP27-induced transcription termination, we asked if sequences upstream of the PAS (UPS) could determine such specificity. We thus cloned the four aforementioned PASs with 1440 nt UPS from the endogenous genes and tested these extended PASs using the pPASPORT assays. Again, ICP27 strongly inhibited mRNA 3′ processing at the extended *SPTSSA* and *KDM4C* PAS (Fig. 5b). By contrast, ICP27 now failed to inhibit mRNA 3′ processing at the extended *POLR2A* PAS. Strikingly, ICP27 even activated mRNA 3′ processing at the extended ICP27 PAS by over 2-fold (Fig. 5b). We performed 3′ RACE to confirm that, although ICP27 inhibited the usage of the core ICP27 PAS, it stimulated its usage when UPS was included (Supplementary Fig. 6). These data suggest that the UPS plays an important role in determining how ICP27 influences mRNA 3′ processing.

Next, we tested if the UPSs alone are sufficient to determine the mode of ICP27 action. To this end, we generated chimeric PAS between *KDM4C* and ICP27, in which the UPSs and the PAS were exchanged (Fig. 5c). Although ICP27 inhibited 3′ processing at the core and extended *KDM4C* PASs (Fig. 5a–b), it had no significant effect when the *KDM4C* PAS was fused to ICP27 UPS (Fig. 5c). Conversely, ICP27 inhibited mRNA 3′ processing at the core ICP27 PAS but functioned as an activator for the extended ICP27 PAS (Fig. 5a–b). When the *KDM4C* UPS was fused to the ICP27 core PAS, this chimeric PAS was strongly inhibited by ICP27 (Fig. 5c), similar to the extended *KDM4C* PAS itself. Therefore, UPS plays a critical role in determining the bimodal activities of ICP27.

### ICP27-RNA interactions modulate its effect on 3′ processing

How do UPSs determine ICP27 activities? It has been proposed that ICP27 binds to GC-rich sequences in the viral mRNAs^37^. The GC content of the ICP27 UPS was substantially higher than that of the *KDM4C* UPS (70.5% vs. 44.1%) (Fig. 5c). This is a general feature of viral vs. cellular UPS (68.3% vs. 40.1%) (Fig. 5d). Additionally, we compared the sequences of host genes that displayed significant DoTT in HSV-1-infected cells to those without significant defects. PAS and surrounding sequences of host genes without DoTT had significantly higher GC content than those with termination defect (Fig. 5e, *p* value = 8.8e−38 by K-S test). These data provide evidence that there is an anti-correlation between the GC-content of PAS and their susceptibility to ICP27-mediated inhibition. We thus hypothesized that ICP27 does not inhibit mRNA 3′ processing when bound near a PAS.

**Fig. 6:**
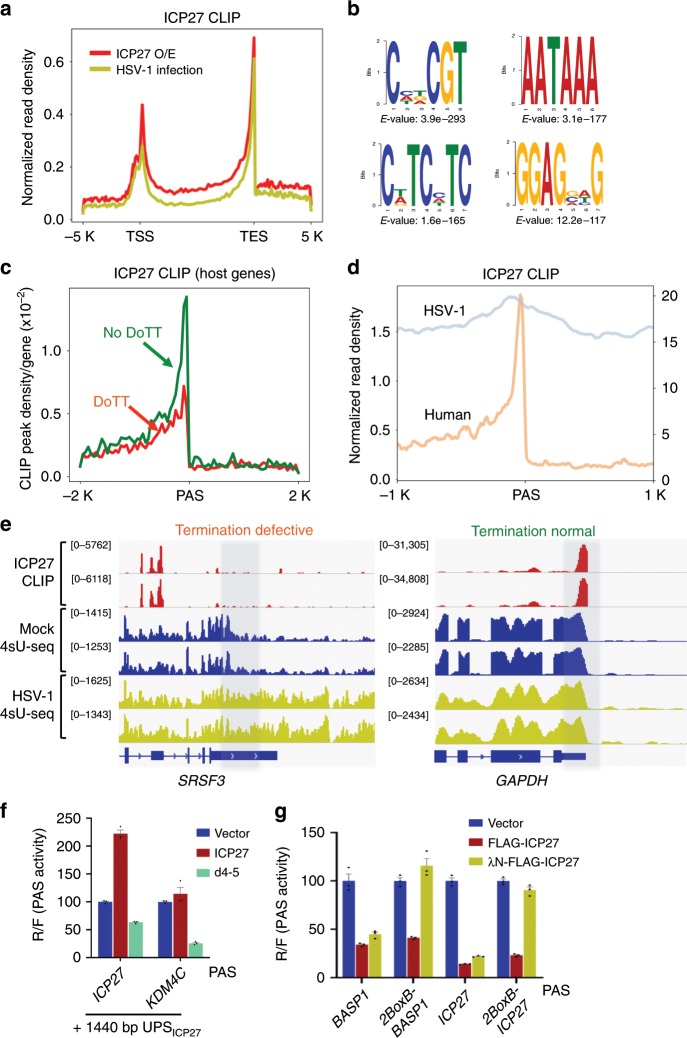
The bimodal activities of ICP27 are regulated by ICP27–RNA interactions. **a** Global distribution of ICP27 CLIP-seq signals along cellular genes. TSS: transcript start site. TES: transcript end site. **b** ICP27 CLIP-seq peak regions were analyzed by DREME and examples of the top enriched motifs are shown. **c** ICP27 CLIP-seq peak densities at PAS of host genes with DoTT (red) and without DoTT (green). **d** ICP27 CLIP-seq peak densities at the PASs of HSV-1 and host genes. **e** ICP27 CLIP-seq tracks at *SRSF3* and *GAPDH* genes. For comparison, 4sU-seq tracks in mock- and HSV-1-infected cells were also shown. The region where termination normally occurs is shaded. **f** Wild-type or d4-5 (ΔRGG) mutant ICP27 or control (empty vector) were co-expressed with pPASPORT constructs containing *ICP27* or *KDM4C* PAS fused to the ICP27 UPS. PAS activities (Renilla/firefly ratio) were plotted as mean ± s.d. **g** Protein tethering assay. Two tandem copies of BoxB hairpin are inserted upstream of the PASs. λN-tagged proteins will be tethered to RNAs via interactions with BoxB. pPASPORT constructs containing PASs labeled on the *x* axis were co-transfected with vector or Flag-ICP27 or λN-Flag-ICP27 expressing plasmids. mRNA 3′ processing efficiencies (Rluc/Fluc) at the PAS are plotted on the *y* axis as mean ± s.d.

To test this hypothesis, we mapped the global ICP27-RNA interaction landscape by CLIP-seq (UV crosslinking and immunoprecipitation followed by high throughput sequencing)^38^. We performed this analysis both in HSV-1-infected and ICP27-overexpressing (O/E) cells (Supplementary Fig. 7). We found that ICP27 preferentially binds cellular mRNAs close to PAS or TESs and, to a lesser degree, at transcription start sites (TSS) (Fig. 6a). Motif analysis of ICP27 RNA binding sites revealed an enrichment of GC-rich sequences (Fig. 6b), consistent with previous in vitro studies^37^. Additionally, AATAAA was enriched in ICP27-associated sequences, consistent with the observed strong ICP27–RNA interactions near PAS/TES (Fig. 6a). These results suggest that ICP27 preferentially binds to GC-rich UPS of PAS.

We next determined whether ICP27 differentially binds to PAS of host genes with or without HSV-1-induced DoTT. While low levels of ICP27–RNA interactions were detected near the PAS of genes with DoTT (Fig. 6c, red line), ICP27 CLIP-seq signals were detected more frequently at PAS without DoTT (Fig. 6c, green line). As exemplified by the *SRSF3* gene that displayed strong DoTT in HSV-1-infected cells (Fig. 1d, 6e), no significant ICP27 CLIP signal was detected near the *SRSF3* PAS (Fig. 6e, left panel). The fact that ICP27 CLIP signals were observed in the 5′ quarter of the mRNA excludes low expression levels or low sensitivity to be responsible for the lack of signal near PAS. By contrast, *GAPDH* showed little or no DoTT in HSV-1-infected cells, but strong and selective ICP27–RNA interactions were detected upstream of its PAS (Fig. 6e, right panel). Together our 4sU-seq and CLIP-seq analyses demonstrate that ICP27 preferentially binds to PASs in genes without DoTT (Fig. 6c).

Since HSV-1 specifically blocks transcription termination of host genes without affecting viral genes, we compared the ICP27–RNA interactions at host and viral PASs. As shown in Fig. 6d, the overall ICP27 CLIP signals (from −1 kb to +1 kb relative to the cleavage site, normalized by RNA levels) were significantly higher at viral PASs than at host PASs, suggesting that ICP27 predominantly binds to viral transcripts. This is consistent with the higher GC-content of the viral genome (Fig. 5d). The anti-correlation between ICP27–RNA interaction and HSV-1-induced DoTT suggests that, once bound to the UPS of a PAS, ICP27 does not induce DoTT.

We directly tested this concept in the following three experiments. First, we synthesized a 100-nt RNA fragment from the UPS of ICP27 (GC content: 63%), which showed no DoTT in HSV-1-infected cells, and another fragment from *KDM4C* (GC content: 41%), which displayed strong DoTT following HSV-1 infection (Fig. 2a). In vitro gel mobility shift assays demonstrated that recombinant ICP27 strongly binds to the GC-rich ICP27 RNA fragment while much weaker binding was observed for the *KDM4C* fragment (Supplementary Fig. 8a). Next we inserted one or two tandem copies of these RNA fragments upstream of a human PAS that is inhibited by ICP27 and tested how these fragments modulate the inhibitory effect of ICP27 using pPASPORT assay (Supplementary Fig. 8b). The ICP27 UPS RNA fragment alleviated ICP27-mediated inhibition of mRNA 3′ processing while the *KDM4C* UPS fragment had no effect (Supplementary Fig. 8b), suggesting that ICP27 interactions with RNA sequences upstream of PASs alleviate ICP27-mediated inhibition of mRNA 3′ processing.

Secondly, we tested if the RNA-binding domain of ICP27, the RGG box, is necessary for its activation function. We repeated the reporter assay as described in Fig. 5c with either the WT ICP27 or the d4-5 mutant, which lacks the RGG box. Consistent with our earlier results, ICP27 activated the extended ICP27 PAS and had little effect on the *KDM4C* fused to the ICP27 UPS (Fig. 6f). Interestingly, however, the d4-5 mutant failed to activate the extended ICP27 PAS and even inhibited both PAS (Fig. 6f), strongly suggesting that the activation function of ICP27 requires its RGG domain and therefore its RNA-binding activity.

Finally, we tested our model using a tethering assay. We inserted two copies of the BoxB hairpin upstream of a host gene (*BASP1*) or ICP27 core PAS (172 nt and 121 nt upstream of the cleavage site, respectively) in pPASPORT (Supplementary Fig. 8c). We then co-expressed these reporters with an empty vector, Flag-ICP27, or λN-Flag-ICP27-expressing plasmids. The expression of Flag-ICP27 significantly repressed mRNA 3′ processing at both PASs (Fig. 6g), consistent with our earlier results (Fig. 4b, 5a). By contrast, the expression of λN-Flag-ICP27, which was tethered to the BoxB-containing PAS through its interactions with the BoxB sequences^39^, had no inhibitory effect (Fig. 6g), suggesting that interactions with RNA prevent ICP27 from inhibiting mRNA 3′ processing. Taken together, we conclude that ICP27 is a general inhibitor of mRNA 3′ processing by disrupting the assembly of a functional mRNA 3′ processing complex. However, by interacting with GC-rich RNA sequences upstream of the PAS, it can activate mRNA 3′ processing, most likely by recruiting the CPSF complex to its target mRNAs.

## Discussion

**Fig. 7:**
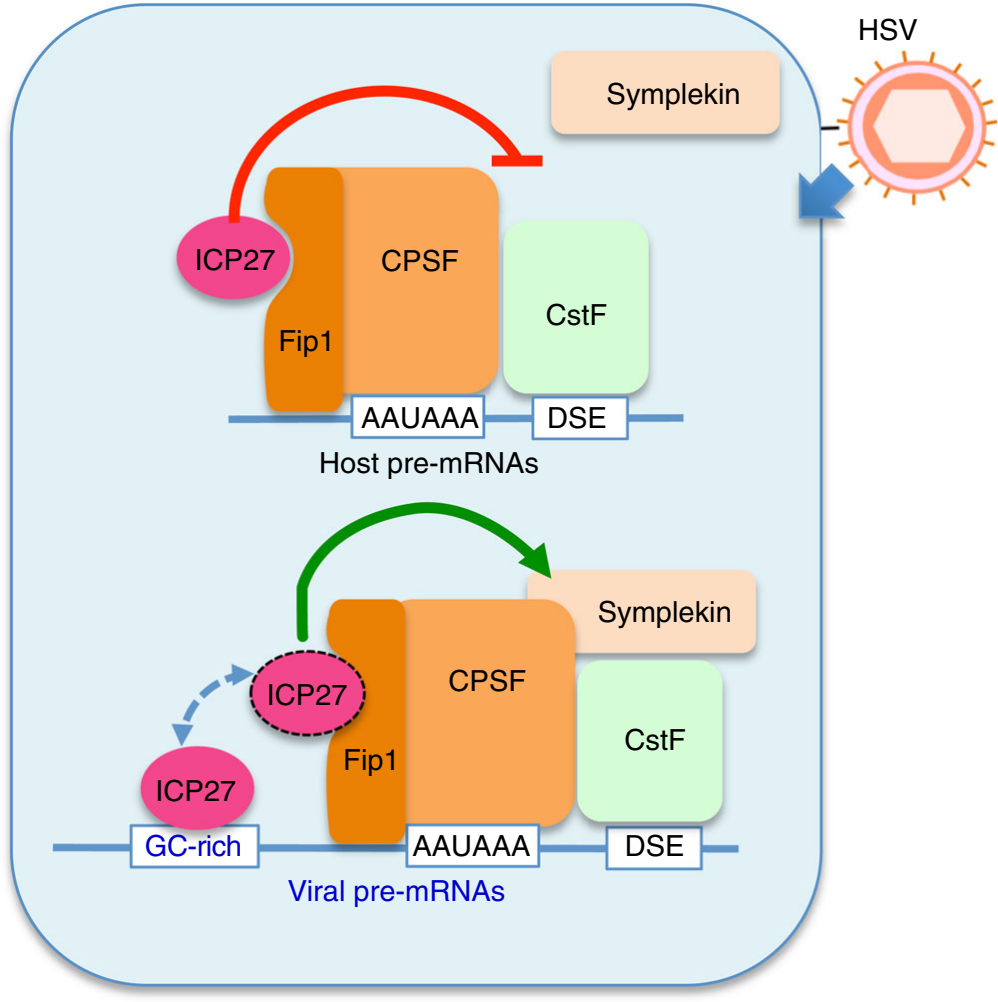
A model for ICP27-mediated regulation of mRNA 3′ processing. In HSV-1-infected cells, ICP27 interacts with CPSF and induces the assembly of an aberrant 3′ processing complex. At viral PASs or host PASs that have GC-rich upstream sequences (UPS), ICP27 binds to these UPS sequences and promotes recruitment of CPSF, thereby activating mRNA 3′ processing. The transition of ICP27 between the CPSF-bound and UPS RNA-bound states is illustrated by a dotted line.

Recent studies revealed that transcription termination is regulated by viral infections and cellular stresses^13–15,40^, and that increased DoTT is associated with cancer^16^. To understand the underlying mechanisms, we carried out an in-depth study of HSV-1-induced DoTT. We found that the HSV-1 immediate early protein ICP27 is sufficient for inducing DoTT when ectopically expressed in human cells. Mechanistically, ICP27 causes DoTT by inducing the assembly of an aberrant mRNA 3′ processing complex via its interactions with the CPSF complex and blocking/delaying mRNA 3′ cleavage. Importantly, ICP27 can also function as a sequence-dependent activator of 3′ end processing, thereby explaining efficient transcription termination of viral transcripts (Fig. 7). It does so through binding to GC-rich sequences upstream of PAS and most likely promoting recruitment of CPSF and other mRNA 3′ processing factors. As transcription termination significantly impacts mRNA production and the subsequent translation^13^, the bimodal activity of ICP27 plays a major role in HSV-1-mediated host shutoff.

mRNA 3′ processing factors are also targeted by other viruses. For example, influenza virus infection or ectopic expression of the influenza viral protein NS1 causes transcription termination defects of host genes^40,41^. NS1 inhibits host mRNA 3′ processing by specifically binding to the CPSF subunit CPSF30 (ref. ^42^). CPSF contains an RNA-binding module (CPSF30, Wdr33, CPSF160, and Fip1) and a cleavage module (CPSF73, CPSF100, and symplekin)^43–45^. As CPSF30 directly binds to the poly(A) signal, AAUAAA, NS1–CPSF30 interaction blocks PAS recognition^43,44,46,47^. Here, we showed that the HSV-1 protein ICP27 directly binds to both modules of CPSF. In contrast to the influenza virus mechanism, however, ICP27 does not block CPSF–RNA interaction and, instead, induces the formation of a “dead-end” 3′ processing complex to block RNA cleavage. In addition to viruses, cellular stress can also modulate the activities of mRNA 3′ processing factors. For example, PAP has been shown to undergo poly(ADP-ribosyl)ation (PARylation) upon heat shock and this post-translational modification inhibits polyadenylation^48^. However, as PAP is not required for transcription termination^12^, PAP poly(ADP-ribosyl)ation is unlikely to mediate heat shock-induced DoTT/DoGs.

Currently the mechanism for stress-induced DoTT/DoGs is unknown. Given the significant overlaps in the genes displaying DoTT/DoGs upon viral infection and cellular stress^15^, we speculate that the mechanism described in this report may have broad implications for understanding stress-induced DoTT/DoG. For example, we showed that ICP27 binds to CPSF and partially displaces symplekin (Figs. 3–4). Interestingly, previous studies have characterized symplekin as a heat labile factor^49^. Therefore, it is possible that heat shock and other cellular stress may lead to degradation or inactivation of symplekin, which in turn results in a failure in mRNA 3′ processing and transcription termination. Additionally, the heat shock transcription factor 1 (HSF1) binds to symplekin upon heat shock and this interaction may promote the mRNA 3′ processing of heat shock protein genes^50^. Therefore, symplekin as well as other mRNA 3′ processing factors may be involved in both the general inhibition and specific activation of transcription termination in cells infected with virus or under stress.

Finally, it should be pointed out that herpesviruses vary in their impact on transcription termination and their ICP27 homologs. First, both murine and human cytomegalovirus do not induce DoTT based on published RNA-seq datasets^51,52^. Second, HSV-1 and HSV-2 are the only human herpesviruses with a high GC content (~70%). The other human herpesviruses, VZV, HCMV, EBV, and KSHV, do not have a high GC content (<60%). Third, although ICP27 is conserved among herpes viruses, ICP27 homologs in HCMV (UL69), EBV (SM protein), and KSHV (ORF57) do not have the same RNA-binding domain (RGG box) and their RNA binding specificities have not been fully defined^53,54^. Taken together, these results suggest that herpesviruses are quite divergent and further studies are needed to fully elucidate their interactions with RNAs and host factors as well as in their impact on transcription termination.

## Methods

### Cell culture, viruses, and infection

Human foreskin fibroblasts (HFF, #86031405, purchased from ECACC) were cultured in DMEM, high glucose, pyruvate (Thermo Fisher #41966052) supplemented with 1× MEM non-essential amino acids (Thermo Fisher #11140050), 1 mM additional sodium pyruvate (Thermo Fisher #11360070), 10% (v/v) fetal bovine serum (FBS, Biochrom #S0115), 200 IU/ml, penicillin (pen), and 200 µg/ml streptomycin (strep). HEK293 and HeLa cell lines were cultured in Dulbecco’s modified Eagle medium (DMEM) with 10% FBS. All cells were incubated at 37 °C in a 5% (v/v) CO_2_-enriched incubator. Virus stocks for WT HSV-1 strain 17 as well as the null mutants of vhs^55^, ICP0 (FXE, strain 17)^56^, ICP22 (R325, strain F)^57^, and TsK (strain 17)^58^ were produced on baby hamster kidney (BHK) cells as described^13^. The temperature-sensitive mutant Tsk was grown at 31 °C. Infections were subsequently performed at 39 °C to render ICP4 inactive. Stocks of the ICP27 null mutant were produced on complementing Vero 2-2 cells^59^. Null mutants of multiple immediate early genes (d100, d103, d106, d107) were a kind gift of Neal DeLuca, grown on complementing cells lines and titrated as described^23,24^. Cells were infected with an MOI of 10 unless otherwise specified and incubated at 37 °C until cells were harvested at the specified time points.

### qRT-PCR

Total RNA was isolated using Trizol reagent following the manufacturer’s instructions. Reverse transcription was performed using All-in-One cDNA Synthesis Supermix (Biotool) including a mix of hexanucleotide random primers and poly-dT primers. qRT-PCR was performed using the SYBR Green 2x Mastermix (Biotool) (qRT-PCR primer sequences in Supplementary Data 2). Relative quantitation was performed using the ΔΔCT approach comparing relative transcript levels downstream and upstream of the PAS.

### 3′ RACE

Total RNA was isolated using Trizol from pPASPORT-transfected cells and 1 μg of total RNA was used to perform reverse transcription using 3′RACE-RT primer and M-MLV (Promega) in 20 μl reaction. cDNA was diluted by five times and 1 μl was used to do first round PCR with 3′RACE-R1, Renilla-F1, and Firefly-F1 primers in one reaction. A total of 1 μl of PCR product from first round PCR was used to carry out the second round PCR with primers 3′RACE-R2, Renilla-F2, and Firefly-F2. PCR products were resolved on 2% agarose gels.

### Reporter assay

PAS sequences were cloned into the multiple cloning sites in pPASPORT. A total of 0.1 μg of reporter constructs were co-transfected with 0.1 μg of ICP27-expressing plasmid into HeLa cells using Polyethylenimine (PEI). Cells were harvested 36 h.p.i. and the Fluc/Rluc ratio was determined using the Dual Luciferase Assay Kit (Promega). For λN Tethering assay, empty vector or ICP27-expressing plasmids and 2× BoxB-BASP1-pPASPORT were co-transfected and the luciferase activities were measured by using the same method. All reporters assays were carried out with three replicates.

### In vitro cleavage assay

All PASs were cloned into pBluescript II KS+ vector, and the RNA substrates were synthesized by in vitro transcription with T7 polymerase in the presence of [α-^32^P]-UTP. In vitro assays for coupled cleavage/polyadenylation assay were performed with HeLa NE as previously described^28^.

### Protein purification

The ICP27 cDNA was cloned in pGEX-4T-1 for GST-ICP27 and pET-51b(+) for Strep-ICP27. MBP-ICP27, GST-ICP27, and Strep-ICP27 fusion proteins were expressed in *Escherichia coli* and purified using amylose beads (NEB), glutathione sepharose (GE Healthcare), and Strep-Tactin® Sepharose (IBA Lifesciences), respectively. Fip1, CPSF73, and CstF cDNAs were cloned into pFastBac. The pFastBac and MultiBac constructs were used to produce recombinant baculoviruses using standard procedures. Baculoviruses were used to infect Sf9 cells and cells were harvested 2 days post-infection. Recombinant proteins were purified with Cobalt beads per manufacturer’s instructions (Fisher).

### 4sU-seq

4sU was added to 5 ml of medium at 500 μM for each 10 cm dish and incubated for 1 h before cells were harvested. For HSV1-infected cells, 4sU was added at 7 h post infection. One hour later, medium was discarded and cells were lysed using 3 ml of Trizol (Invitrogen). Total RNAs were isolated from the cells following the manufacturer’s instructions. A total of 700 μl of thiol-specific biotinylation reaction contained 70 μg of total RNA, 70 μl of 10× Biotinylation Buffer (100 mM Tris-Cl pH 7.5 and 10 mM EDTA), 140 μl of biotin-HPDP (in 1 mg/ml DMF), and 70 μl of DMF. This was incubated at room temperature for 1.5 h with rotation. Excessive Biotin-HPDP was removed by chloroform extraction twice. RNA was precipitated, washed once with 75% ethanol, and re-suspended in 100 μl of H_2_O. Biotinylated RNA samples were heated to 65 °C for 10 min and immediately placed on ice. A total of 100 μl of pre-washed streptavidin-magnetic beads were washed with 800 μl of 1× washing buffer (5 mM Tris-Cl pH 7.5, 0.5 mM EDTA, and 1 M NaCl) for 3 × 5 min and incubated with 100 μl of 0.1 M DTT for 2 × 5 min at room temperature; 1/10 the volume of 5 M NaCl and an equal volume of isopropanol and 1 μl of glycoblue were added to precipitate RNA.

### CLIP-seq

We used a modified version of eCLIP. Briefly, infected and transfected HeLa cells were exposed to 254 nM UV with 400 mJ/cm^2^, and then scrapped and re-suspended in lysis buffer (50 mM Tris-HCl pH 7.4, 100 mM NaCl, 1% NP-40, 0.1% SDS, 0.5% sodium deoxycholate, and protease inhibitor). After RNase I digestion, 80 μl of protein G Dynabeads beads and 5 μg of ICP27 antibody per IP were used for immunoprecipitation overnight. After washing with high salt buffer (50 mM Tris-HCl pH 7.4, 1 M NaCl, 1 mM EDTA, 1% NP-40, 0.1% SDS, and 0.5% sodium deoxycholate) and wash buffer (20 mM Tris-HCl pH 7.4, 10 mM MgCl_2_, and 0.2% Tween-20), phosphates at 5′ and 3′ end of RNA were removed by CIP (NEB) for 15 min at 37 °C. 3′ RNA linker was added by T4 RNA ligase (Thermo Fisher Scientific) overnight and [γ-^32^P]ATP (PerkinElmer) was used to label the 5′ end of RNA by T4 PNK (NEB). ICP27/RNA complex was resolved in 4–12% NuPAGE (Invitrogen) and transferred to NC membrane. Proper regions of membrane were cut out to extract RNA in PK/Urea buffer. Extracted RNA was added with 5′ RNA linker by T4 RNA ligase and reverse-transcribed with Superscript III (Invitrogen). Libraries for CLIP-seq were generated by PCR using Illumina universal primer and index primers.

### Immunoprecipitation and western blotting

Cells in 10 cm dishes were harvested by trypsinization and washed once with PBS. Cells were then re-suspended in 1 ml of IP buffer (150 mM NaCl, 10 mM Tris-HCl pH 7.4, 1 mM EDTA pH 8.0, 1% Triton X-100, and 0.5% NP-40) containing 1× protease inhibitor and 10 μg of RNase A/T1, and rotated for 10 min at 4 °C followed by sonication. Cell lysates were cleared by centrifugation for 20 min at 4 °C and the supernatant was transferred to a new 1.7-ml tube. Three micrograms of ICP27 P1119 antibody and 30 μl of protein G magnetic beads (30 μl of anti-FLAG M2 affinity gel for FLAG-ICP27-transfected cells) were added in cell lysate and incubated for 4 h at 4 °C. Wash the beads with 4 × 1 ml of IP buffer × 10 min, and then 50 μl of 1× SDS loading buffer was added to the beads and heated for 5 min at 95 °C. Samples were analyzed by SDS-PAGE and western blotting. The antibodies for western blotting are as follows: ICP27 (Virusys, P1113 and P1119), CPSF160 (Bethyl, A301-580A), CPSF100 (Bethyl, A301-581A), CPSF73 (Bethyl, A301-091A), CPSF30 (Bethyl, A301-585A), Fip1 (Bethyl, A301-462A), Symplekin (Bethyl, A301-465A), WDR33 (Bethyl, A301-152A), CstF64 (Bethyl, A301-092A), CstF77 (Bethyl, A301-096A), CstF50 (Bethyl, A301-250A), CFIm68 (Bethyl, A201-358A), CFIm59 (Bethyl, A301-360A). The dilutions for all antibodies are 1:2000.

### Gel shift assay

[α-^32^P]-UTP-labeled RNA was incubated with 1 mM ATP, 20 mM creatine phosphate, 100 ng/μl yeast tRNA, and 44% HeLa NE in 10 μl reaction at 30 °C for 20 min. The reactions were cooled on ice and heparin was added to 0.4 μg/μl; 6 μl of the reaction was resolved on 4% native PAGE in 1× Tris-Glycine running buffer at 100 V for 210 min in cold room and visualized by phosphor imaging.

### Glycerol gradient sedimentation and RNA pulldown

A total of 10 pmol of 3MS2-ICP27pA RNA was incubated with 500 pmol of MBP-MS2 fusion protein on ice for 30 min, and then mixed with 1 mM ATP, 20 mM creatine phosphate, 100 ng/μl yeast tRNA, and 200 μl of HeLa NE in 500-μl reaction with/without 25 μg of GST-ICP27. The reaction mix was incubated at 30 °C for 20 min followed by chilling on ice. Heparin was added to 0.4 μg/μl. The whole reaction was performed on 10–30% glycerol gradient sedimentation at 30k rpm for 15 h, and fractions were collected manually from top at 0.5 ml/fraction; 20 μl of pre-washed amylose beads were mixed with each fraction and rotated for 1 h at 4 °C. Beads were washed with wash buffer (20 mM HEPES-KOH (pH 7.9), 100 mM KCl, 1 mM MgCl_2_, 1% Triton X-100, and 0.5 mM DTT) for 3 × 10 min and then the complexes were eluted with 120 μl of wash buffer plus 12 mM maltose for 2 × 20 min at 4 °C. Eluted proteins were precipitated with acetone overnight at −20 °C and analyzed by SDS-PAGE followed by silver staining or western blotting.

### Data analysis

4sU-seq data in Fig. 1 and Supplementary Fig. S1 were mapped against hg19, human rRNA sequences, and HSV-1 strain 17 using ContextMap v2.7.9. Other sequencing data were mapped to human genome (hg19) and/or HSV KOS using STAR version 2.5.2a (ref. ^60^). No multimapping was allowed in alignment. Bigwig files were then generated using deepTools v3.0.2 with RPKM normalization.

CLIP data were pre-processed by Cutadapt, and the adapter sequence or any tails of “A”s were trimmed prior to the alignment. For these data, peaks were called using GEM^61^ with the suggested setting for CLIP data. Peak calling was done for each strand separately and then the results were merged.

Read distribution data around termination sites or gene body were generated using deepTools. Peak distribution data were extracted by Homer software^62^. The visualization and any post-processing step then were done in Python.

Termination defect was studied based on the ratio of reads mapped on 5 kb downstream of the termination site to the expression of the gene. This ratio (termination ratio) was calculated for each gene and in all conditions, and its change was used as the measure of termination defect. Bedtools^63^ was used to extract the downstream region, and featureCounts^64^ generated the read counts. The read counts were normalized by the length of the genes. Genes were then ranked based on the change in termination ratio and then based on their coverage and defected and not defected ones were chosen from this list. We applied a threshold of 5-fold change for the termination ratio and 5 reads per kilo basepairs for coverage to find the significant changes in termination. Genes with a small change in their termination ratio (<1.1 fold) were marked as the ones with no significant change.

Bedtools was used to extract the sequence for each region under study when necessary. Any post processing then was done in Python. Motif analysis was done using DREME^65^.

### Reporting summary

Further information on research design is available in the Nature Research Reporting Summary linked to this article.

## Supplementary information

Supplementary Information

Reporting Summary

Description of Additional Supplementary Files

Supplementary Data 1

Supplementary Data 2

## Data Availability

4sU-seq and CLIP-seq data have been deposited to the GEO database (accession number: GSE128753). The mass spectrometry proteomics data were deposited to the ProteomeXchange Consortium via the PRIDE partner repository with the dataset identifier PXD012837. The Source Data for Figs. 3b–d, 4a, e, f, and Supplementary Figs. 2a, b, 3a, b, 4a, b, and 6–8 are provided as a Source Data file.

## Source data

Source Data
