# Supplementary material for: Herpes simplex virus blocks host transcription termination via the bimodal activities of ICP27
Source: Nat Commun. 2020 Jan 15;11:293. doi: 10.1038/s41467-019-14109-x (PMC6962326; doi:10.1038/s41467-019-14109-x)
Supplement: Supplementary file 1 — Supplementary Information [file 41467_2019_14109_MOESM1_ESM.pdf]

Herpes simplex virus blocks host transcription termination via the bimodal activities of ICP27

Xiuye Wang, Thomas Hennig, Adam W. Whisnant, Florian Erhard, Bhupesh Prusty, Caroline C. Friedel, Elmira Forouzmand, William Hu, Luke Erber, Yue Chen, Rozanne M. Sandri-Goldin, Lars Dölken, Yongsheng Shi

Supplementary information

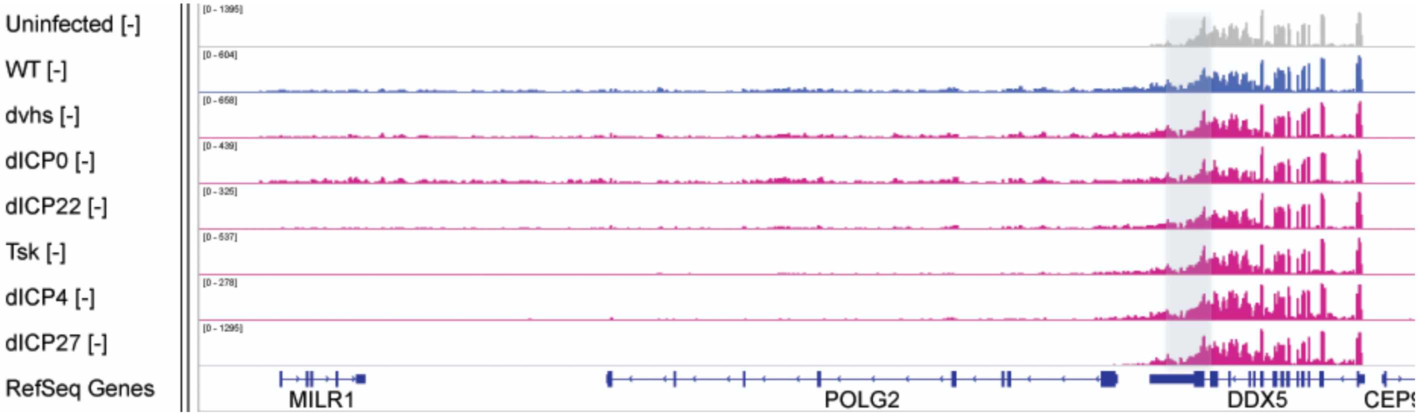

Supplementary Fig. 1 (related to Fig. 1d). Mapped 4sU-seq reads for *DDX5* in cells infected with wild-type or various mutant HSV-1 strains. The region where transcription termination occurs in mock-infected cells is shaded.

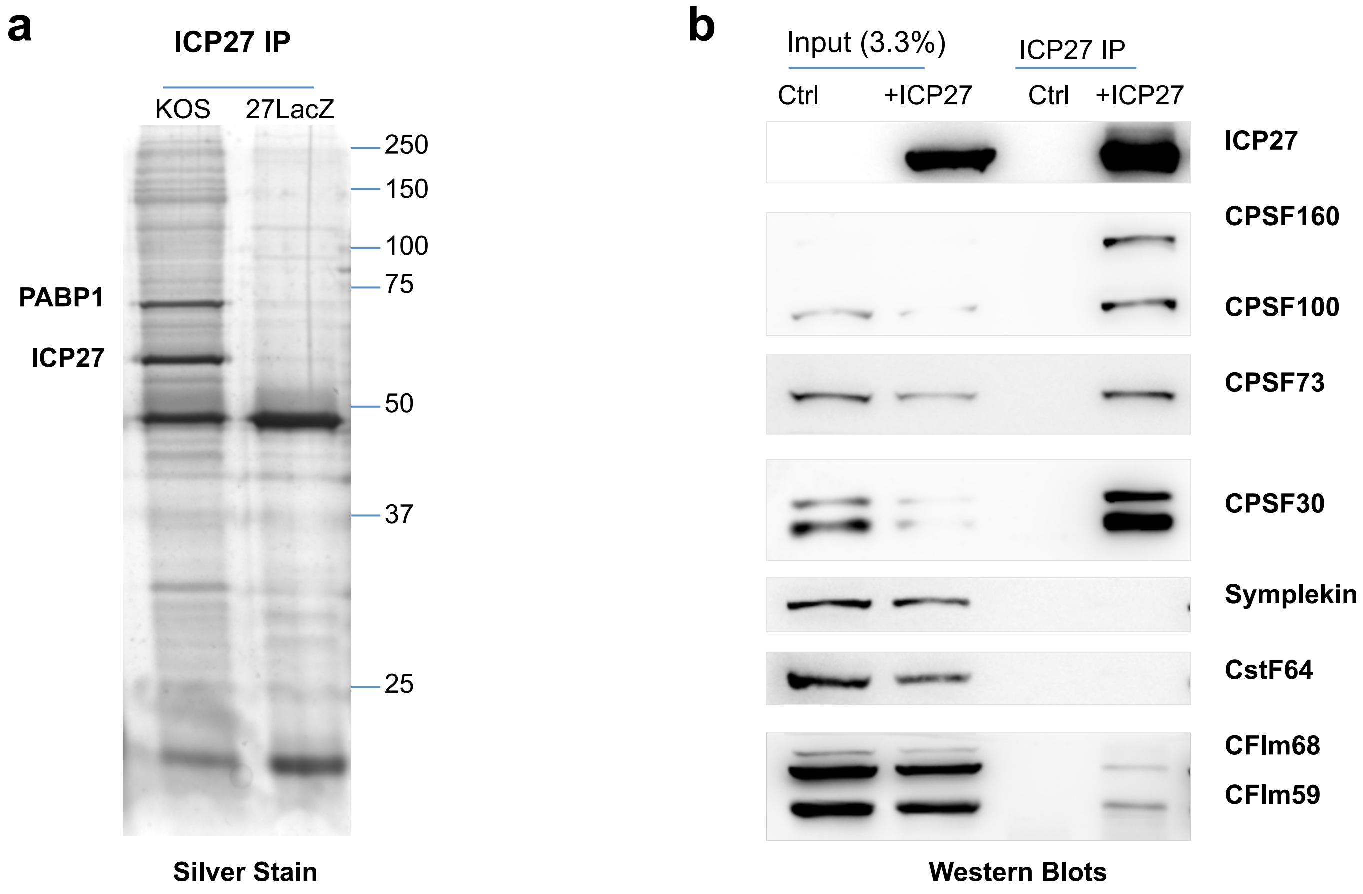

Supplementary Fig. 2 (related to Fig. 3). (a) Lysates from HeLa cells infected with wild-type (KOS) or ICP27 deletion mutant (27lacZ) HSV-1 were used for immunoprecipitation with anti-ICP27 antibody. IP samples were resolved by SDS-PAGE and visualized by silver staining. **(b)** HeLa cells transfected with control (empty vector) or an ICP27-expressing plasmid were used for IP with an anti-ICP27 antibody. IP samples were analyzed by western blotting. Source data are provided as a Source Data file.

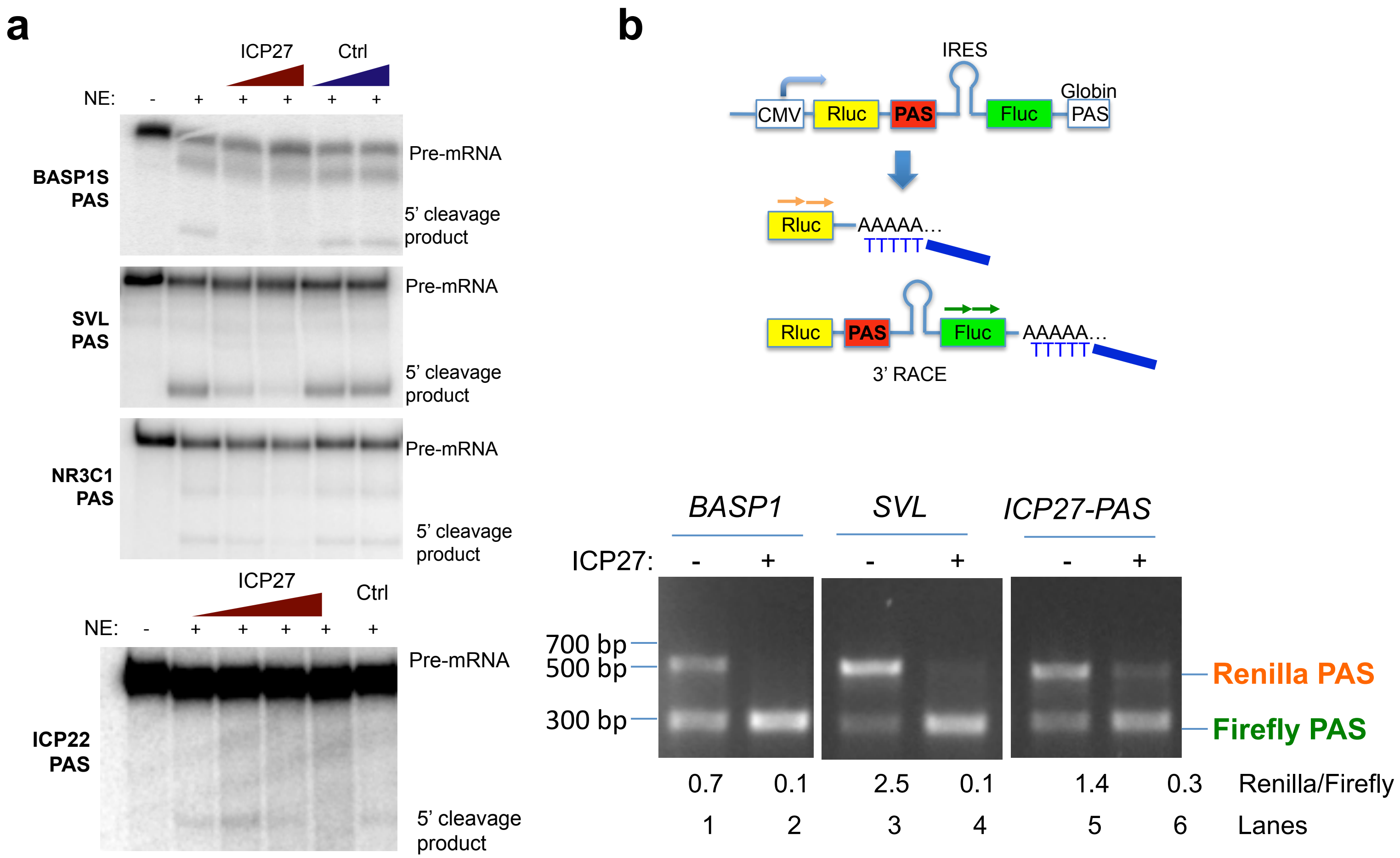

Supplementary Fig. 3 (related to Fig. 4a). (a) In vitro cleavage assay with HeLa nuclear extract (NE) using the specified PAS pre-mRNA substrate. 5' cleavage product is marked. Different amounts of recombinant MBP-ICP27 (labeled as ICP27) or MBP-MS2 (Ctrl) were added to NE. **(b)** Top panel: The pPASPORT construct gives rise to two RNA isoforms. 3' RACE (nested PCR with two primers) was performed using an oligo-dT-linker primer, a Rluc-specific primer and a Fluc-specific primer. PCR products were resolved on agarose gels and visualized by SYBR green staining (lower panel). Data quantification results are shown below the gel images. Source data are provided as a Source Data file.

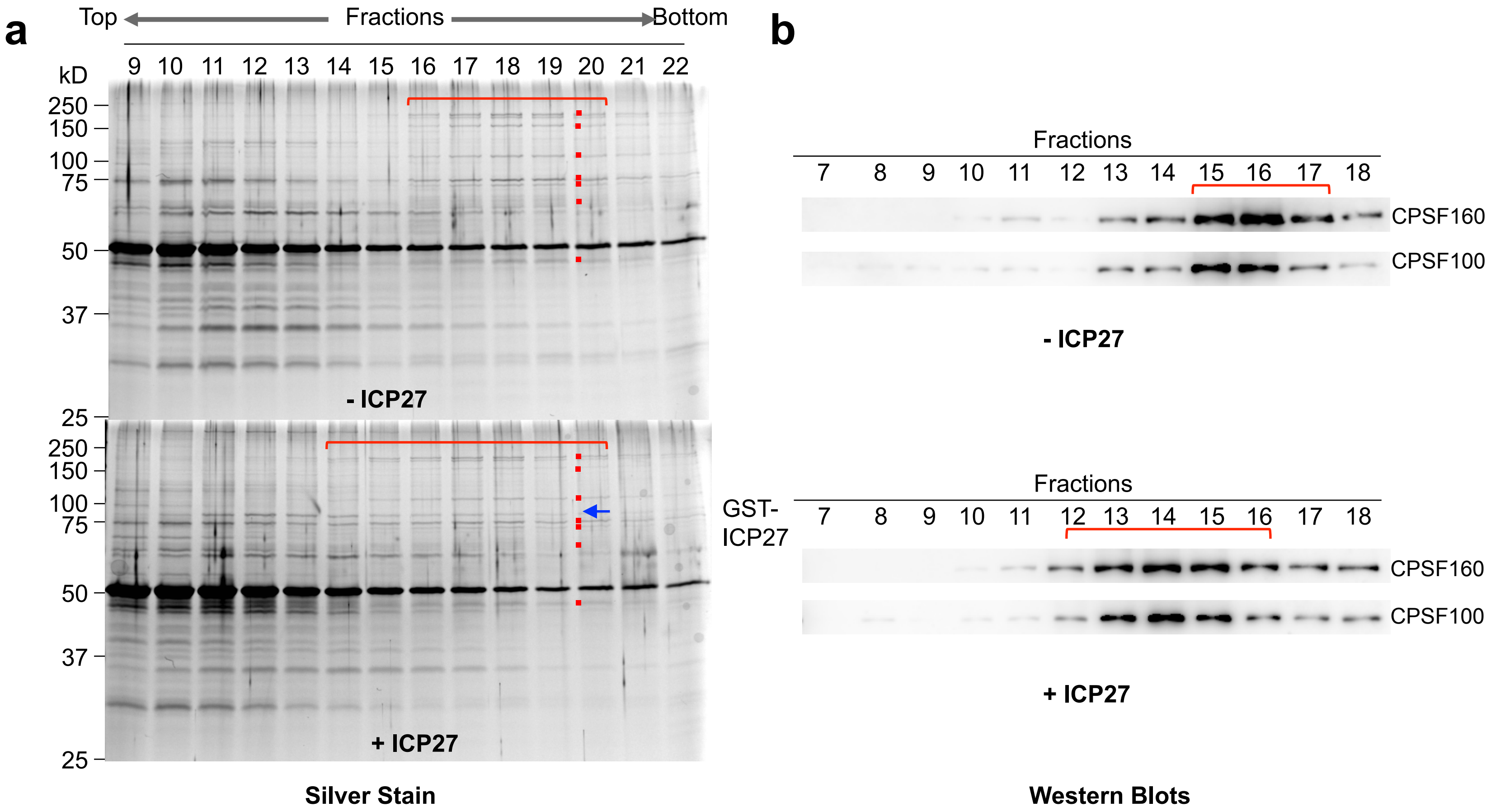

Supplementary Fig. 4 (related to Fig. 4e-f). mRNA 3' processing reactions in the absence (-ICP27) or presence (+ICP27) were loaded onto a 10-30% glycerol gradient. Fractions were resolved by SDS-PAGE and visualized by silver staining (left panels) or western blotting (right panels). The peak fractions containing the mRNA 3' processing complex (P complex) were marked by red brackets. Protein bands corresponding to known 3' processing factors are marked by red dots. ICP27 bands are marked by blue arrows.

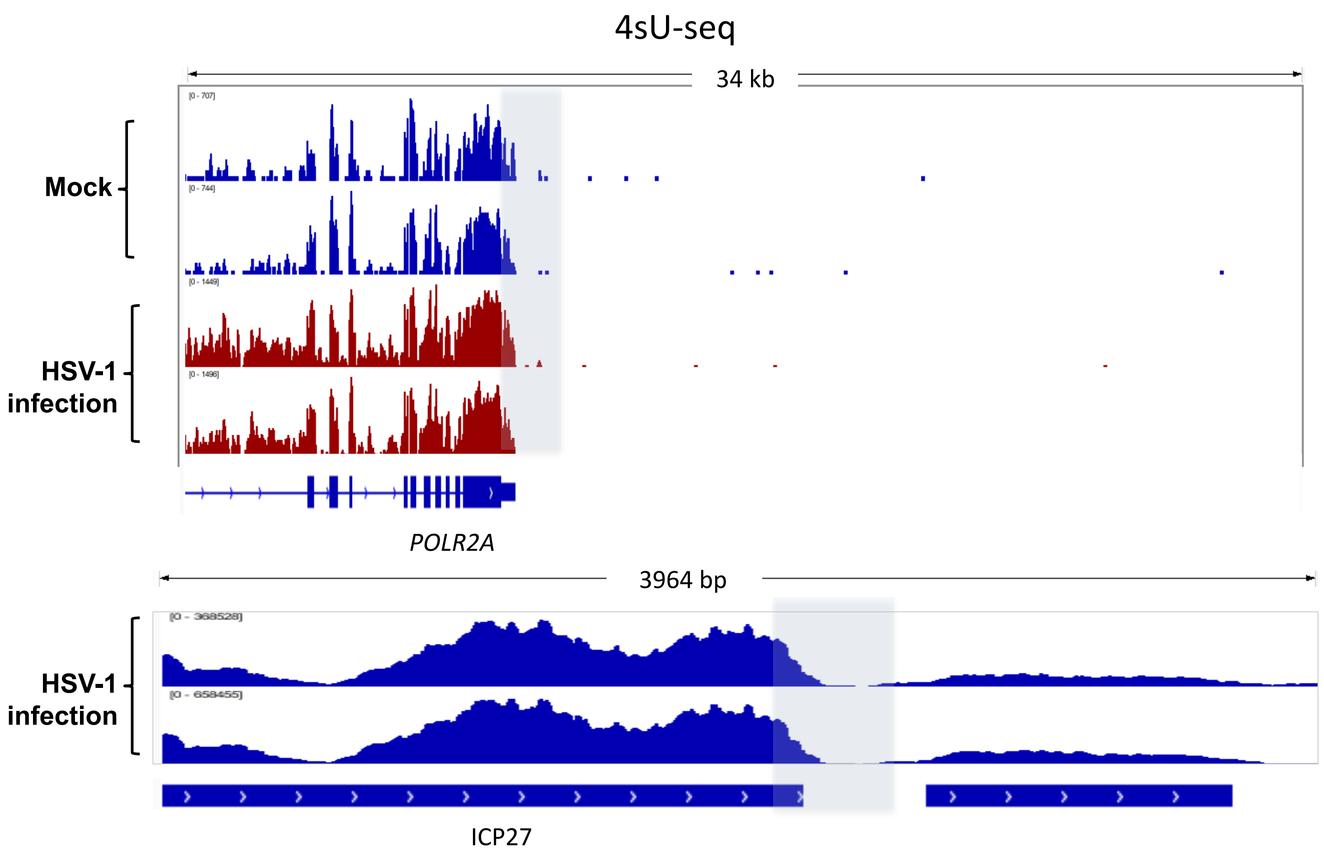

Supplementary Fig. 5 (related to Fig. 5). Mapped 4sU-seq reads for *POLR2A* in mock infected cells or cells infected with HSV-1 (top panel) and 4sU-seq reads for *ICP27* in HSV-1-infected cells (bottom panel). The region where transcription termination occurs in mock-infected cells is shaded.

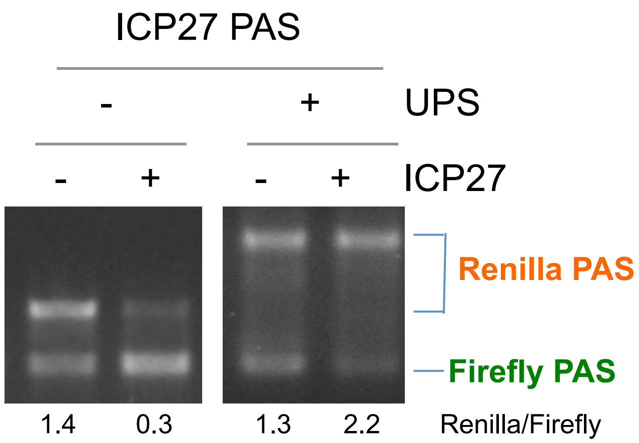

Supplementary Fig. 6 (related to Fig. 5b). 3' RACE (similar to Supplementary Fig. 3b) of the RNAs isolated from cells expressing pPASPORT containing ICP27 PAS with or without upstream sequences (UPS), and with or without over-expression of ICP27 protein. The Renilla PAS PCR products are larger due to the insertion of UPS. The left panel (also shown in Supplementary Fig. 3b) was included for comparison. Source data are provided as a Source Data file.

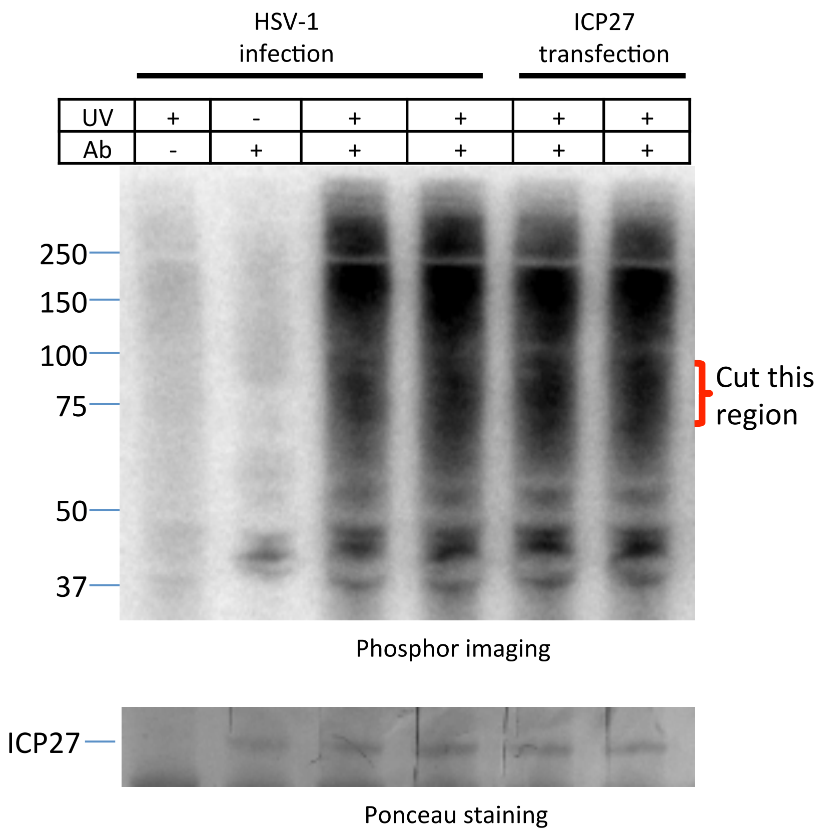

Supplementary Fig. 7 (related to Fig. 6). HeLa cells infected with HSV-1 or transfected with an ICP27-expressing plasmid were UV irradiated and the cell lysates were used for IP with an anti-ICP27 antibody. The immunoprecipitated protein-RNA complexes were resolved by PAGE and visualized by phosphorimaging (RNAs were 5' labeled). The marked regions were cut out for RNA extraction. Ponceau staining were performed (lower panel) to ensure equal amounts of ICP27 were immunoprecipitated. Source data are provided as a Source Data file.

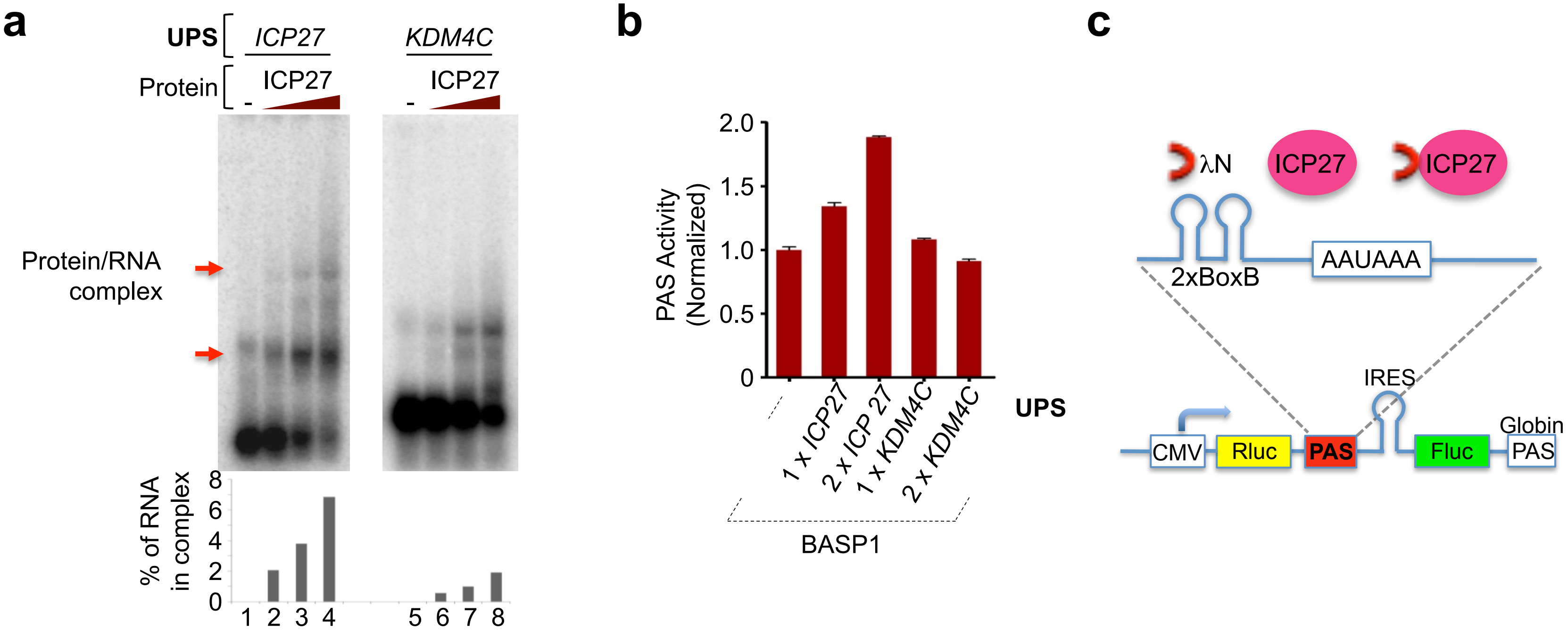

Supplementary Fig. 8 (related to Fig. 6). (a) The UPS fragments from ICP27 and *KDM4C* genes were synthesized in vitro and used in gel mobility shift assays with increasing concentrations of recombinant ICP27. Bands corresponding to protein-RNA complexes are marked by red arrows. **(b)** One (1x) or two (2x) copies of the UPS fragments from ICP27 and *KDM4C* genes were inserted upstream of the *BASP1* PAS in pPASPORT. These constructs were transfected with or without co-transfection of ICP27. PAS activities (Rluc/Fluc) were measured and normalized (+ICP27/-ICP27) and plotted as mean ± s.d . **(c)** Reporter for the tethering assay shown in Fig. 6g. Two tandem copies of BoxB hairpin were inserted 50 nt upstream of the cleavage site in L3 PAS, which in turn was inserted into pPASPORT. λN-ICP27 or ICP27 were co-expressed with the reporter. Source data are provided as a Source Data file.
