# Supplementary material for: Herpes simplex virus blocks host transcription termination via the bimodal activities of ICP27
Source: Nat Commun. 2020 Jan 15;11:293. doi: 10.1038/s41467-019-14109-x (PMC6962326; doi:10.1038/s41467-019-14109-x)
Supplement: Supplementary file 3 — Description of Additional Supplementary Files [file 41467_2019_14109_MOESM3_ESM.pdf]

Description of Additional Supplementary Files

File Name: Supplementary Data 1
Description: contains mass spectrometry analysis results

File Name: Supplementary Data 2
Description: contains information on the oligos, plasmids, antibodies used in this study
